# Typhus Fever in Paris

**Published:** 1842-10-01

**Authors:** 


*Typhus Fever in Paris.*—For six weeks or two months, typhus fever has raged in Paris to as great or greater extent than it did in the year 1831. It is not confined to the hospitals, where several wards are filled with persons suffering under it, but it is extensively spread throughuot the city, in the middle classes. There being none of the usual causes of such epidemics present, in the distress of the lower classes, or the bad nature of the food, it has been attributed, though perhaps with insufficient reason, to the dry and hot weather which has prevailed for three or four months. The disease follows pretty much its ordinary train of symptoms, with the exception that many of the patients present a marked bilious state, and have a great disposition to vomit bile, which is excited by the least movement or cough. But the course of the affection appears to undergo no great change from the developement of these phenomena; and it is said that in spite of the number of persons severely affected, the average of mortality is not greater than is usual in this disease.—*Lond. Med. Gaz.*

